# The Protective Role of Feruloylserotonin in LPS-Induced HaCaT Cells

**DOI:** 10.3390/molecules24173064

**Published:** 2019-08-23

**Authors:** Yuzhu He, Byung-gook Kim, Hye-Eun Kim, Qiaochu Sun, Shuhan Shi, Guowu Ma, Young Kim, Ok-su Kim, Ok-joon Kim

**Affiliations:** 1 Department of Oral Pathology, School of Dentistry, Chonnam National University, Gwangju 61186, Korea; 2 Department of Oral and Maxillofacial Surgery, School of Stomatology, Dalian Medical University, Dalian 116044, China; 3 Department of Oral Medicine, School of Dentistry, Chonnam National University, Gwangju 61186, Korea; 4 Department of Orthodontics, School of Dental Medicine, University of Pennsylvania, Philadelphia, PA 19104, USA; 5 Department of Periodontology, School of Dentistry, Chonnam National University, Gwangju 61186, Korea

**Keywords:** feruloylserotonin, epidermal inflammation, HaCaT cell, anti-inflammation, anti-oxidation

## Abstract

Epidermal inflammation is caused by various bacterial infectious diseases that impair the skin health. Feruloylserotonin (FS) belongs to the hydroxycinnamic acid amides of serotonin, which mainly exists in safflower seeds and has been proven to have anti-inflammatory and antioxidant activities. Human epidermis mainly comprises keratinocytes whose inflammation causes skin problems. This study investigated the protective effects of FS on the keratinocyte with lipopolysaccharides (LPS)-induced human HaCaT cells and elucidated its underlying mechanisms of action. The mechanism was investigated by analyzing cell viability, PGE_2_ levels, cell apoptosis, nuclear factor erythroid 2-related factor 2 (Nrf2) translocation, and TLR4/NF-κB pathway. The anti-inflammatory effects of FS were assessed by inhibiting the inflammation via down-regulating the TLR4/NF-κB pathway. Additionally, FS promoted Nrf2 translocation to the nucleus, indicating that FS showed anti-oxidative activities. Furthermore, the antioxidative and anti-inflammatory effects of FS were found to benefit each other, but were independent. Thus, FS can be used as a component to manage epidermal inflammation due to its anti-inflammatory and anti-oxidative properties.

## 1. Introduction

Inflammatory skin lesions caused by various bacterial infectious diseases, such as acne, folliculitis, furuncles, and carbuncles, mainly occur on the epidermis as nodules, pustules, and papules [1,2,3,4]. Reactive oxygen species (ROS) have been found to contribute to epidermal inflammation by damaging the follicular epithelium and inducing melanin deposition [1]. The goal of epidermal inflammation treatment is to control existing lesions and prevent permanent scarring as much as possible [2]. Epidermal inflammation therapies combine systematic and local treatments such as non-steroid anti-inflammatory drugs (NSAIDs), retinoids, antibiotics, hormonal therapy, and other complementary medicines [5]. These medicines generally regulate the epidermal cell growth, reduce hyperpigmentation, and have anti-inflammatory, anti-bacterial, and anti-oxidative effects. In order to achieve a good therapeutic effect, it is essential to use various medicines cooperatively [6]. Therefore, it will be reasonable to search for an alternative drug with both anti-inflammatory and anti-oxidative effects to protect the epidermis from infectious skin diseases, rather than using the current complex drug combinations.

Feruloylserotonin (FS; *N*-feruloyl-5-hydroxy-tryptamine) is isolated from safflower seeds and has been shown to have anti-atherogenic, anti-oxidative, and anti-melanogenesic activities [7,8]. FS is comprised of 5-hydroxytryptamine (5-HT) and ferulic acid which may contribute to its anti-oxidative and anti-inflammatory properties [9]. It has been reported that the combined therapy of FS and methotrexate can reduce inflammation in adjuvant arthritis [10]. Nuclear factor-κB (NF-κB) is an important transcriptional regulator of inflammatory damage that is often considered to be a therapeutic target for inflammatory diseases. FS can inhibit NF-κB activation in human aortic endothelial cells treated with high levels of glucose [11]; however, the anti-inflammatory effects of FS were only proven by detecting its anti-inflammatory efficiency. No detailed mechanism has yet been elucidated for the action of FS against LPS (lipopolysaccharides) induced epidermal inflammation. It has also been found through DCF-DA and XTT (2,3-*bis*(2-methoxy-4-nitro-5-sulfophenyl)-2H-tetrazxolium-5-carboxanilide inner salt) assays that FS can inhibit oxidative stress in response to cytokines, bacterial invasion, and other factors [7]. The anti-oxidative activity of FS can also inhibit H_2_O_2_-induced melanogenesis by down-regulating the microphthalmia-associated transcription factor expression via extracellular signal-regulated kinase phosphorylation [12]. Nrf2 (nuclear factor erythroid 2-related factor 2) is thought to be a master regulator of cellular survival, whose translocation during oxidative stress induces genes to produce antioxidant enzymes. Although Nrf2 is an important regulator that controls many pathways in response to oxidative stress, there is no direct evidence that it is affected by FS. Both NF-κB and Nrf2 are important transcription factors in almost all cell lines that regulate cellular responses to inflammation and oxidation, respectively. Since functional cross-talk exists between these two pathways, specific anti-inflammatory or anti-oxidative functions must be confirmed.

Keratinocytes comprise almost 95% of the cell mass of the human epidermis, the inflammation of which is considered as skin problem [13]. LPS are the main component of Gram-negative bacterial membranes and are one of the most common stimulators of inflammation [14]. In this study, we established an epidermal bacterial infection inflammatory model in human keratinocyte HaCaT cells treated with LPS. In order to determine the effects of FS on epidermal inflammation, the anti-inflammatory and anti-oxidative properties of FS was assessed in LPS-induced HaCaT cells. Evidence was provided that FS not only inhibits the Toll-like receptor 4-mediated nuclear factor-κB (TLR4/NF-κB) pathway, but also promotes the translocation and stability of Nrf2 by analyzing cell ability, prostaglandin E2 (PGE_2_) release, ROS production, cell apoptosis, TLR4/NF-κB-related protein expression, and Nrf2 translocation. FS can be used as a component of topical drugs to manage inflammatory skin lesions in infectious diseases because of its anti-oxidative and anti-inflammatory properties.

## 2. Results

### 2.1. LPS and FS on HaCaT Cells

**Figure 1:**
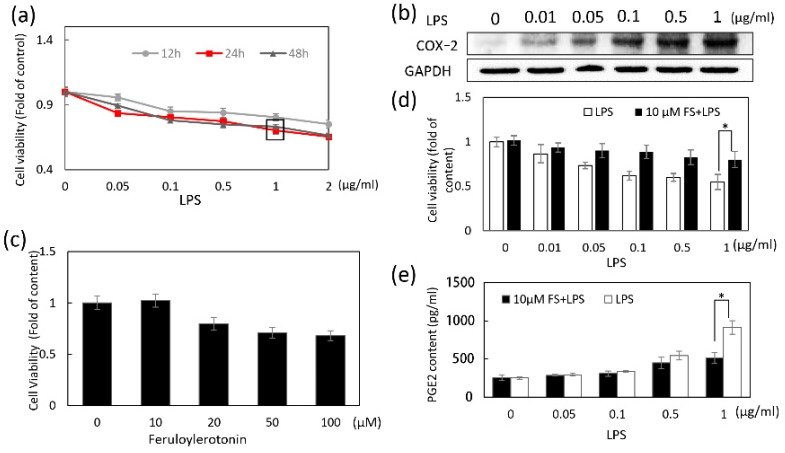
The effects of lipopolysaccharides (LPS) and feruloylserotonin (FS) on HaCaT cells. Cell viability was analyzed by MTT (3-(4,5-dimethylthiazol-2-yl)-2,5-diphenyltetrazolium bromide). (**a**) HaCaT cells were treated with 0–2 µg/mL LPS for 12, 24, and 48 h. (**b**) COX-2 levels in 0–1 µg/mL LPS-induced HaCaT cells were detected with Western blotting. (**c**) The HaCaT cells were treated with 0–100 µM FS for 24 h. (**d**) The cell viabilities in only LPS group and FS-LPS group were compared under the treatment of 0 to 1 µg/mL LPS. (**e**) The released prostaglandin E2 (PGE_2_) levels in only LPS group and FS-LPS group were measured with the prostaglandin E_2_ assay kit. * *p*-value < 0.05.

Cell viability was analyzed by MTT (3-(4,5-dimethylthiazol-2-yl)-2,5-diphenyltetrazolium bromide). As shown in Figure 1a, the cell viability of HaCaT cells decreased in a time- and dose-dependent manner to approximately 60% when treated with 1 µg/mL LPS for 24 h. The increased level of COX-2 induced by LPS (1 µg/mL for 24 h) indicated that the inflammation happened (Figure 1b). The viability of HaCaT cells treated with 10 μM FS was similar to that of the control group (Figure 1c). Furthermore, the increase of HaCaT cell viability in FS pretreated group (Figure 1d) was more significant compared to cells co-treated (Figure S1) and post-treated (Figure S1) with FS. Therefore, HaCaT cells were pretreated with 10 μM FS followed by 1 µg/mL LPS for 24 h (Figure S2). Under these conditions, cell viability increased significantly compared to the LPS-treated cells (Figure 1d). PGE_2_ level in inflammation was measured using an Elisa kit after 24 h LPS treatment with or without FS pretreatment. PGE_2_ release was induced by LPS treatment and decreased by FS pretreatment (Figure 1e).

### 2.2. The Comparison of Function between FS and NSAIDs

**Figure 2:**
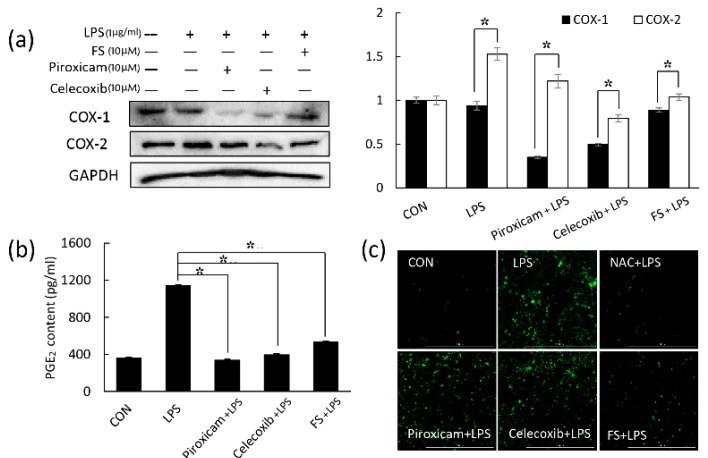
Comparison between FS and COX-1/COX-2 inhibitors in HaCaT cells. (**a**) In order to compare the activity of FS on COX-1 and COX-2, 10 µM piroxicam, 10 µM celecoxib, and 10 µM FS were used to inhibit COX-1 and COX-2 respectively. (**b**) The effects of piroxicam, celecoxib, and FS on PGE_2_ release were detected. (**c**) To compare the anti-oxidative activities of piroxicam, celecoxib, and FS, DCF-DA staining was used to detect the reactive oxygen species (ROS). A total of 5 µM NAC (*N*-acetyl-*L*-cysteine) was used to scavenge ROS as a positive control. * *p*-value < 0.05.

In order to assess the effects of FS on inflammation, the anti-inflammatory activity of FS was compared with piroxicam (COX-1 inhibitor) and celecoxib (COX-2 inhibitor). Western blotting revealed that the expression level of COX-2 in FS pretreated group was higher than that in piroxicam treated group. The effect of FS on COX-1 expression level could not be observed (Figure 2a). PGE_2_ release was clearly decreased by FS, but weaker than the two inhibitors (Figure 2b). In order to measure the anti-oxidative activity of FS and NSAIDs, NAC was added to scavenge the ROS using a positive control (Figure S3). Through observing the total ROS in Figure 2c, the result of FS pretreated group showed the decrease of ROS, but not in piroxicam or celecoxib treated groups. Although the NSAIDs performed better on decreasing the expressions of COX-1 and COX-2, they had no effects on ROS. Thus, the dual functions of FS performed on anti-inflammation and anti-oxidation were benefit to protect LPS-induced keratinocyte.

### 2.3. The Action of FS on TLR4/NF-kB Pathway

**Figure 3:**
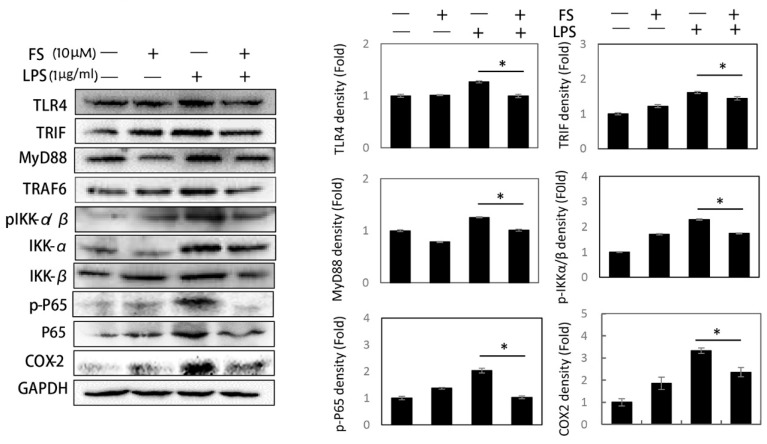
Inhibition of FS on the TLR4/NF-kB pathway. The expressions of proteins on TLR4/NF-κB pathway were detected by Western blotting. The main protein levels in the results of Western blotting were measured by Image J software and shown in the diagram. * *p*-value < 0.05.

Toll-like receptor 4 (TLR4) is a LPS receptor related to the MyD88 and TRIF branches of the NF-κB pathway. Therefore, the protein expression on TLR4/NF-κB pathway were detected by Western blotting. Comparing FS-LPS group to LPS group, although the expression of TRIF was decreased by FS (0.16 fold of control), the decrease of MyD88 (0.25 fold of control) was more significant. The decrease of other main proteins were as follows: TLR4 (0.27 fold of control), pIKKα/β (0.49 fold of control), p-P65 (1.01 fold of control), and COX-2 (0.97 fold of control). The decreased levels of pIKKα/β and p-P65 indicated the anti-inflammatory activity of FS. The inhibitory effect of FS on the proteins of TLR4/MyD88/NF-kB pathway in HaCaT cells alleviated the inflammatory response (Figure 3).

### 2.4. The Effect of FS on ROS and Apoptosis

**Figure 4:**
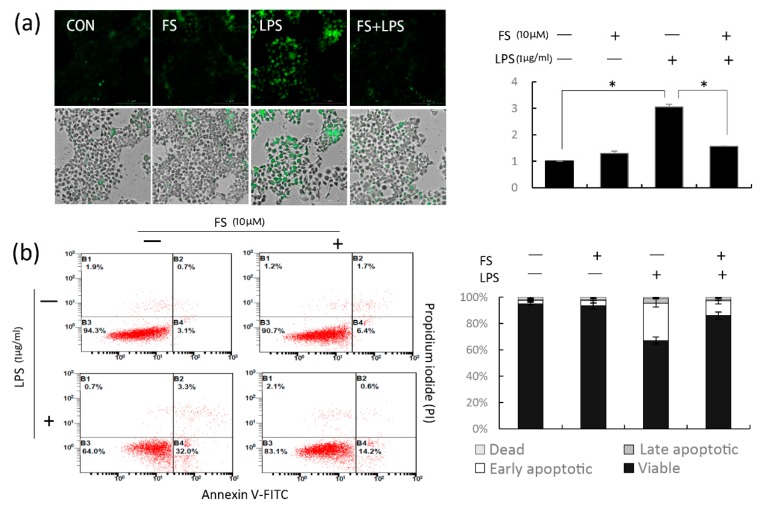
The inhibition of FS on ROS and apoptosis. (**a**) The intracellular ROS was detected with DCF-DA staining. (**b**) To investigate the effect of FS on cell apoptosis, HaCaT cells stained with annexin V/propidium iodide were analyzed by flow cytometry. * *p*-value < 0.05.

As apoptosis was induced by oxidative stress, the anti-oxidative activity of FS was evaluated by measuring the intracellular ROS and cell apoptosis. Green fluorescence in HaCaT cells indicated intracellular ROS localization. When HaCaT cells were treated with LPS, the intensity of fluorescence was higher than that in the control group; however, when the cells were pretreated with FS, LPS-induced ROS levels were decreased (Figure 4a). To evaluate the effect of FS on cell apoptosis, HaCaT cells with or without FS pretreatment were exposed to LPS and analyzed by flow cytometry via annexin V/propidium iodide staining (Figure 4b). Comparing the FS group with the control group, there was no significant difference in the cell apoptosis. Comparing the LPS group to the FS-LPS group, the apoptotic cell ratio decreased from 32% to 14.2%, suggesting that FS pretreatment reduced the LPS-induced apoptosis of HaCaT cells, as observed for the MTT results (Figure 1d).

### 2.5. Effects of FS on the Translocation and Stability of Nrf2

**Figure 5:**
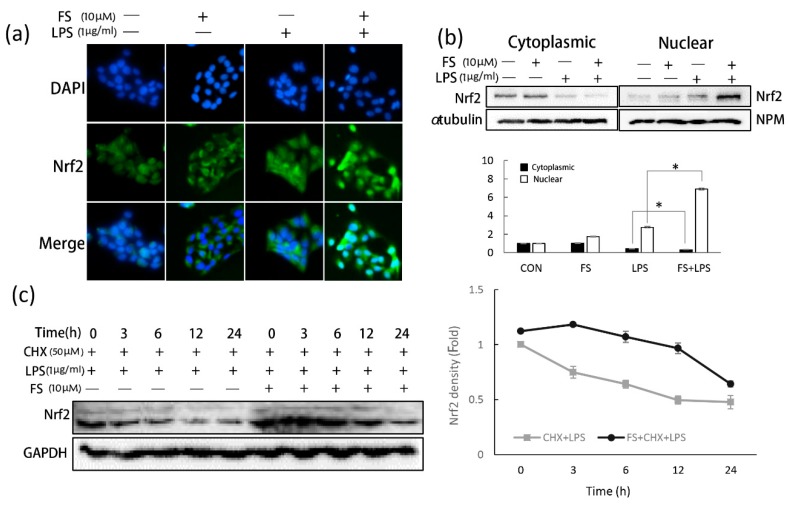
The effects of FS on Nrf2 translocation and stability. (**a**) In order to confirm the translocation of Nrf2, the immunofluorescence staining was used to observe Nrf2 location via green fluorescence. 4‘,6-diamidino-2-2phenylindole (DAPI) was used to show the location of nucleus via blue fluorescence. (**b**) Nuclear Nrf2 (nuclear factor erythroid 2-related factor 2) levels in HaCaT cells were measured with Western blotting. (**c**) 50 µM cycloheximide (CHX) was used to block the synthesis of Nrf2 in HaCaT cells to evaluate the effect of FS on Nrf2 stability. * *p*-value < 0.05.

Immunofluorescence staining was used to detect the location of Nrf2 in HaCaT cells (Figure 5a). When exposed to LPS, Nrf2 fluorescence accumulated in the cytoplasm of the HaCaT cells and slightly found in the nucleus. Nuclear Nrf2 fluorescence intensity in FS-LPS group was much higher than that in the other groups. In the FS-pretreated HaCaT cells, Nrf2 accumulated around the nucleus, but translocation did not occur; however, once the HaCaT cells were exposed to LPS, Nrf2 translocated to the nucleus. These results suggested that FS was not directly responsible for Nrf2 translocation, but promoted it. To confirm this hypothesis, Nrf2 levels in the nucleus and cytoplasm were measured by Western blotting (Figure 5b). Similar to the results shown in Figure 5a, when exposed to LPS, the level of nuclear Nrf2 in HaCaT cells pretreated with FS was significantly higher than that in non-FS pretreated cells. In order to explain the reason that the Nrf2 level in the FS-pretreated group was higher than in the control group, Nrf2 stability was assessed. Cycloheximide (CHX) was used to block Nrf2 synthesis and investigate the role of FS in Nrf2 stability (Figure 5c). It showed that FS increased Nrf2 stability via post translational modification and that LPS decreased Nrf2 levels in a time-dependent manner.

### 2.6. The Action of FS on Inflammation and Oxidation

**Figure 6:**
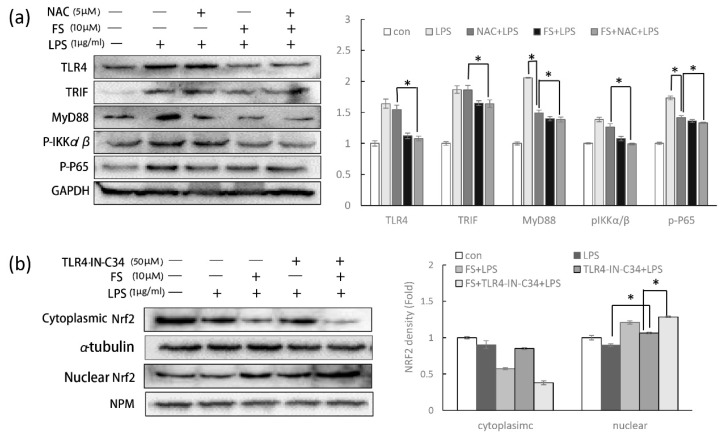
The action of FS under the condition of inflammation or oxidation. (**a**) Scavenging ROS with 5 μM NAC to avoid the effect of oxidation on TLR4/NF-κB pathway. The proteins expressions were measured with Western blotting. (**b**) TLR4-IN-C34 as the inhibitor of TLR4 was used to discard the influence of TLR4 pathway on Nrf2 translocation. The action of FS on Nrf2 translocation was investigated by Western blotting. * *p*-value < 0.05.

It has been reported that LPS-induced ROS can increase cellular inflammation; however, ROS-induced Nrf2 translocation can produce anti-oxidant enzymes to dampen COX2 production [15]. Evading the effect of Nrf2 on inflammation to detect the single action of FS on the TLR4/NF-κB pathway, NAC was used to scavenge ROS. Under the condition of scavenging ROS (inactivating Nrf2 translocation), the TLR4/NF-κB pathway was still inhibited by FS (Figure 6a). ROS had no significant effect on the expression of most TLR4/NF-κB pathway proteins, except for MyD88 and p-P65 whose levels were down-regulated by NAC. Thus, anti-inflammatory property of FS could be enhanced by its anti-oxidative activity, but is not dependent on it.

Cellular Nrf2 activity is regulated by multiple mechanisms, one of which is the NF-κB pathway [15]. In order to detect the anti-oxidative property of FS without the influence of TLR4/NF-κB pathway, TLR4 inhibitor was used. When the TLR4/NF-κB pathway was inhibited by TLR4-C34-IN (TLR4 inhibitor), the Nrf2 translocation was still promoted by FS (Figure 6b). And regardless of FS pretreatment, nuclear Nrf2 expression could be increased by inhibiting TLR4. Therefore, the anti-oxidative property of FS could be enhanced by its anti-inflammatory activity, but not dependent on it.

## 3. Discussion

FS is a compound extracted from safflower seeds that exhibits anti-oxidative and anti-inflammatory properties. Here, we examined these two activities of FS in LPS-induced HaCaT cells.

The viability of HaCaT cells treated with 1 μg/mL LPS for 24 h was approximately 60%; however, the reduction in apoptosis because of FS was relevant in this study. LPS reduced the viability of pretreated cells with or without FS in a dose-dependent manner; however, the decrease was less in the FS-pretreated group than in the group without FS (Figure 1d), indicating that FS pretreatment maintained cell stability. Similarly, flow cytometry result suggested that FS pretreatment reduce the LPS-induced apoptosis of HaCaT cells (Figure 4a).

PGE_2_ plays an important role in generating inflammatory responses and is induced by the inflammatory stimulator COX-2. COX-1 may also be a dominant source of PGE_2_ for housekeeping functions [16,17]. The NSAIDs piroxicam and celecoxib were used to inhibit COX-1 and COX-2, respectively. FS did not affect COX-1 expression compared to piroxicam (Figure 2a); therefore, we focused on COX-2 in this study. Western blotting and a PGE_2_ assay revealed that FS exhibited a weaker anti-inflammatory activity than piroxicam and celecoxib (Figure 2a,b), whereas the ROS assay suggested that FS exhibited better anti-oxidative effects than the two NASIDs (Figure 2c). Thus, FS was found to have the potential to manage epidermal inflammation. LPS-induced COX-2 expression and PGE_2_ release occurred via the TLR4/NF-κB pathway and were both inhibited by FS pretreatment. It has been reported that LPS can induce TLR4 expression and lead to NF-κB pathway activation [18]. The LPS-induced activation of the TLR4/NF-κB pathway was related to the following proteins: TLR4, MyD88, TRIF, TRAF6, pIKK-α/β, IKK-α, IKK-β, p-P65, and P65 (Figure 3) [19]. The FS pretreatment of HaCaT cells inhibited the expression of TLR4/NF-κB pathway proteins, suggesting that FS be beneficial on LPS-induced inflammation. Some protein expressions in FS group as a positive group were higher than that in control group. This difference was inferred to be caused by the basal condition of the HaCaT cells. Once the HaCaT cells were treated with FS, the basal condition was different from control group, followed by the changes of protein levels. In the experiment of detecting TLR4/NF-κB pathway, the difference between LPS group and FS-LPS group was remarkable.

The anti-inflammatory effects of FS were confirmed by analyzing total ROS level (Figure 4a). Nrf2 is thought to be a master regulator of cell survival [20]. Under basal conditions, Nrf2 resides in the cytoplasm and translocates inactively. If cells experience ROS-induced oxidative damage, Nrf2 is actively translocated to the nucleus [21]. FS exerted anti-oxidative effects by stimulating Nrf2 translocation. In LPS-treated HaCaT cells, Nrf2 translocated into the nucleus a little; however, when treated with both FS and LPS, nuclear Nrf2 levels increased greatly. In FS-pretreated HaCaT cells, Nrf2 accumulated around the nucleus; however, translocation did not occur until the HaCaT cells were exposed to LPS (Figure 5a). These results suggest that when pretreated with FS, Nrf2 translocation was directly induced by LPS, with FS acting as a stimulator, concordant with the western blotting results (Figure 5b). It was also shown that cytoplasmic Nrf2 levels were slightly higher in FS-pretreated HaCaT cells than in the control group. To explain this observation, CHX was used to block Nrf2 synthesis and investigate the role of FS on Nrf2 stability (Figure 5c). CHX is often used to block messenger RNA translocation and thus block protein synthesis [22]. Within 24 h, FS increased Nrf2 stability in CHX-blocked HaCaT cells, particularly when exposed to LPS for 3-12 h, indicating that FS prevented Nrf2 ubiquitination and degradation [23].

Furthermore, it has been reported that LPS-induced ROS can increase cellular inflammation, while ROS-induced Nrf2 transcription can produce anti-oxidant enzymes to reduce COX-2 production [24]. To evade the complex relationship described above and detect the specific function of FS in the TLR4/NF-κB pathway, NAC was used to clear LPS-induced ROS. The cleared ROS avoid the effect of Nrf2 translocation on evaluating the action of FS on TLR4/NF-κB pathway. Notably, without the effect of Nrf2 on inflammation, FS still performed the activity on the anti-inflammation (Figure 6a).

The anti-inflammatory role of Nrf2 in the TLR4/NF-κB pathway has been discussed above; however, it should be noted that NF-κB can also regulate Nrf2 activity. It has been well established that CREB-binding protein is a transcriptional co-activator of both Nrf2 and NF-κB, with NF-κB overexpression thought to limit Nrf2 complex formation by competing with CBP (CREB-binding protein) [15]. In order to eliminate the effect of the TLR4/NF-κB pathway on Nrf2 translocation, TLR4 inhibitor was used. The Nrf2 translocation in HaCaT cells pretreated with FS and TLR4-IN-C34 was still promoted, suggesting that the promotion of FS on Nrf2 translocation is independent from its anti-inflammatory property (Figure 6b). Nuclear Nrf2 level in the TLR4-C34-IN pretreated HaCaT cells were slightly lower than the group without TLR4-C34-IN, suggesting that Nrf2 translocation was slightly regulated by the activity of TLR4/NF-κB pathway.

In this study, the mechanism of FS on anti-inflammation and anti-oxidation was investigated via TLR4/NF-κB pathway and Nrf2 translocation. Although the inhibition of FS on TLR4/NF-κB pathway was proved, its target needs to be confirmed. FS has both anti-inflammatory and anti-oxidative properties compared to the existing NSAIDs, as shown in our results. Further investigation is required to see if FS can be used in clinical applications.

In summary, FS exhibited anti-inflammatory and anti-oxidative activities in LPS-induced human keratinocyte HaCaT cells by inhibiting the TLR4/NF-κB pathway and promoting Nrf2 translocation. These two properties benefited each other, but independently; thus, FS has the potential of being a component to manage epidermal inflammation because of its anti-inflammatory and anti-oxidative properties.

## 4. Materials and Methods

### 4.1. Chemicals and Antibodies

LPS was purchased from Sigma (Sigma-Aldrich, St. Louis, MO, USA). Specific antibodies for Nrf2, NPM, α-tubulin, GAPDH, COX2, TLR4, MyD88, TRAF6, and p-IKKα/β were obtained from Santa Cruz Biotechnology (Santa Cruz, CA, USA). Antibodies for TRIF, IKK-α, IKK-β, NF-kB (P65), and pNF-kB (p-P65) were purchased from Cell Signaling Technology (Cell Signaling Technology, Beverly, MA, USA). FS was synthesized by reacting activated hydroxycinnamic acid esters with serotonin hydrochloride under alkaline conditions.

### 4.2. Cell Culture

HaCaT cells were cultured in Dulbecco’s Modified Eagle’s Medium (DMEM) supplemented with 10% fetal bovine serum (FBS; JR Scientific, Woodland, CA, USA) and penicillin/streptomycin (Wel gene, Gyeongsan, Korea) at 37 °C in a humid 5% CO_2_ atmosphere for 24 h. Cells were then pretreated with FS at the indicated concentration for 24 h and exposed to LPS (Sigma-Aldrich, St. Louis, MO, USA) for 24 h. To detect the effects of FS on Nrf2 stability, HaCaT cells were pretreated with or without FS for 24 h and 50 µM cycloheximide (CHX, Sigma-Aldrich, St. Louis, MO, USA) for 4 h. To inhibit the TLR4/NF-kB pathway, cells were pretreated with 50 µM TLR4 inhibitor (TLR4-IN-C34, Sigma-Aldrich, St. Louis, MO, USA) before LPS. To clear ROS, cells were pretreated with 5 µM *N*-acetyl-*L*-cysteine (NAC, Sigma-Aldrich, St. Louis, MO, USA) before LPS. To compare the anti-inflammatory activity of FS with NSAIDs, COX-1 inhibitor (Piroxicam) and COX-2 inhibitor (Celecoxib) were used to pretreat the HaCaT cells at a concentration of 10 µM. (The cells without any treatment: control group; only FS-treatment: FS group; merely LPS-treatment: LPS group; FS pretreatment and LPS treatment: FS-LPS group).

### 4.3. Cell Viability Assay

HaCaT cells (1 × 10^3^ cells/well) were seeded in 96-well plates (Corning, NY, USA) with DMEM containing 10% FBS and 1% penicillin and streptomycin at 37 °C in a humid 5% CO_2_ atmosphere and treated with 0, 10, 20, 50, or 100 µM/mL FS for 24 h. MTT reagent (3-(4,5-dimethylthiazol-2-yl)-2,5-diphenyltetrazolium bromide (Sigma-Aldrich, St. Louis, MO, USA) was added to each well and incubated for a further 4 h. The MTT reagent was discarded, cells were incubated with DMSO for 10 min, and their absorbance was measured at 570 nm using a microplate reader (Bio-rad, Hercules, CA, USA). Viable cell percentage was calculated as follows: cell viability = mean absorbance in test well/mean absorbance in control well. The same method was used to assess the viability of HaCaT cells pretreated with 10 µM/mL FS and 0, 10, 50, 100, 500, or 1000 ng/mL LPS.

### 4.4. PGE_2_ Release Assay

HaCaT cells were pretreated with or without FS and treated with 0, 50, 100, 500, or 1000 ng/mL LPS for 24 h. All supernatants were then collected and their PGE_2_ levels were measured using a commercially available ELISA kit (R & D Systems, Minneapolis, MN, USA) with a microplate reader.

### 4.5. Cellular Reactive Oxygen Species (ROS) Detection

Total ROS production in HaCaT cells was determined by the 2′,7′-dichlorodihydrofluorescein diacetate (DCF-DA, Sigma-Aldrich, St. Louis, MO, USA) fluorescence method. Briefly, HaCaT cells (approximately 70% confluence in a 6-well plate) were incubated at 25 °C in PBS with DCF-DA away from light for 20 min. The PBS containing DCF-DA was then aspirated and cells were rinsed twice with cold PBS. The fluorescence of oxidized DCF-DA in the cell lysates, an index of ROS formation, was measured by fluorescence microscopy with 488 nm excitation and 530 nm emission filters (Lionheart^TM^ FX, Winoski, VT, USA).

### 4.6. Apoptosis Analysis

LPS-induced HaCaT cell apoptosis was analyzed by Annexin V-FITC/propidium iodide (PI) (Invitrogen, Carlsbad, CA, USA) staining. Appropriately treated HaCaT cells were harvested and washed in cold PBS, centrifuged, and re-suspended in annexin-binding solution to which a working solution of FITC-annexin V and PI was added. After 15 min incubation at 25 °C, the samples were immediately analyzed under single laser emitting excitation by flow cytometry.

### 4.7. Immunofluorescence Staining

To analyze intracellular Nrf2 localization, HaCaT cells were grown as polarized monolayers on 12 mm tissue culture inserts (Nunc, NY, USA), fixed with 4% paraformaldehyde in PBS for 20 min at 25 °C, and incubated with 0.1% Triton X-100 in PBS for 15 min. The cells were then incubated in 1% BSA-PBS for 1 h at 25 °C and with anti-Nrf2 antibodies (1:100 in 1% BSA-PBS, m/v) overnight at 4 °C. Cells were washed three times with 1% BSA-PBS followed by staining with goat anti-rabbit IgG secondary antibodies. And the nucleus was stained with diluted DAPI (Molecular Probes). The stained cells were mounted and imaged using a laser confocal scanning microscope (Lionheart FX, Winoski, VT, USA).

### 4.8. Western Blotting Analysis

At the indicated time points, HaCaT cells were lysed with modified radioimmunoprecipitation assay (RIPA) buffer (50 mM Tris-Cl, 150 mM NaCl, 1% Nonidet P-40, and 0.1% SDS) with PMSF and a protease inhibitor cocktail. Extracted proteins were subjected to 7.5% SDS-PAGE gel electrophoresis and transferred to polyvinylidene difluoride (PVDF) membranes (Millipore) followed by being blocked with 2% milk-TBS buffer at 25 °C for 1 h. After being blocked, the membranes were probed with primary antibodies at 4 °C overnight using the following antibodies related to the TLR4/NF-κB pathway and Nrf2: TLR4 (1:1000), MyD88 (1:500), TRIF (1:1000), TRAF6 (1:500), pIKK-α/β (1:1000), IKK-α (1:1000), IKK-β (1:1000), pNF-κB (1:1000), NF-κB (1:1000), COX-2 (1:500), GAPDH (1:1000), Nrf2 (1:1000), NPM(1:1000), and α-tubulin (1:1000). After being washed with TBST four times, the membranes were incubated with secondary anti-rabbit IgG (sc-2004) or anti-mouse IgG (sc-2005) antibodies for 2 h at 25 °C and visualized using an enhanced chemiluminescence kit.

### 4.9. Statistical Analysis

All the results were expressed as mean ± standard deviation. The statistical analysis was performed by one-way ANOVA test using SPSS software. All data were repeated at least three times. If *p* < 0.05, the result was considered significant difference.

## Supplementary Materials

The following are available online, Figure S1: The cell survival by FS co-treatment and FS post-treatment. Figure S2: Time table for cell treatment. Figure S3: The cell viability of NAC and the ROS intensity (DCF-DA staining). Figure S4: The structure of FS and schematic diagram of the pathways.
