# Supplementary figures and images for: The Protective Role of Feruloylserotonin in LPS-Induced HaCaT Cells
Source: Molecules. 2019 Aug 23;24(17):3064. doi: 10.3390/molecules24173064 (PMC6749254; doi:10.3390/molecules24173064)

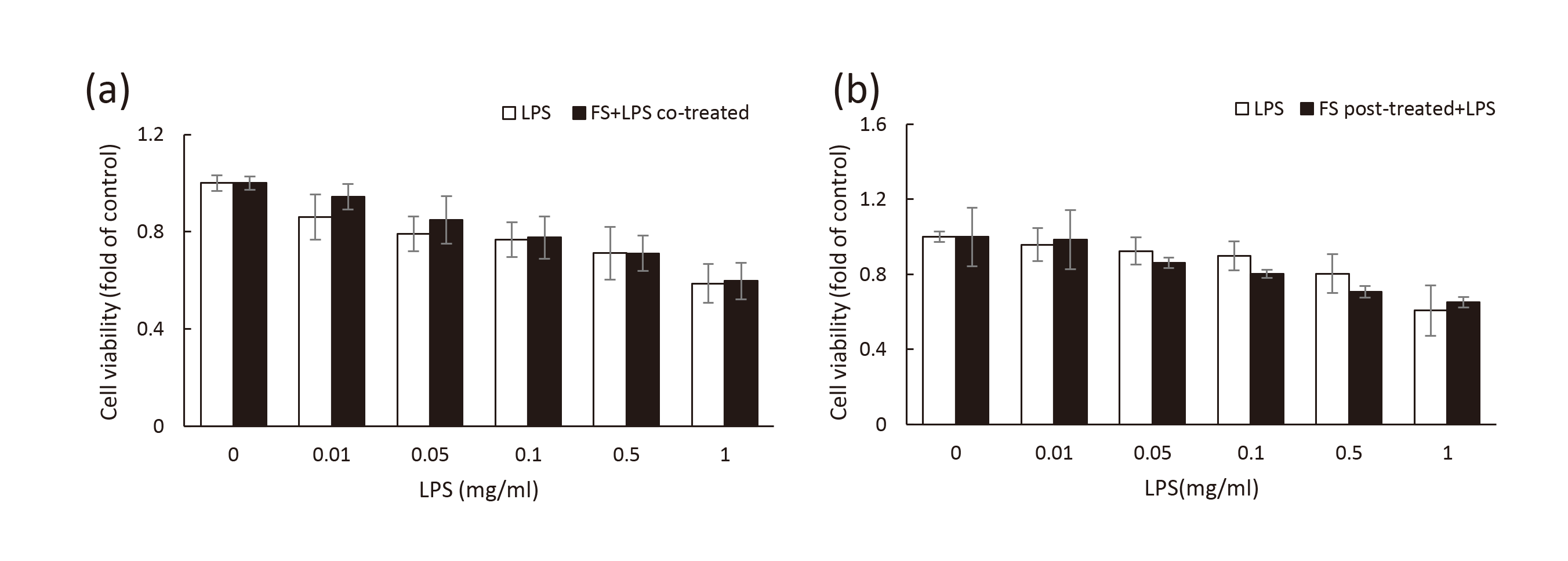

Supplement: Supplementary file 1 [file molecules-24-03064-s001.zip › Supplementary File/Figure S1.tif]

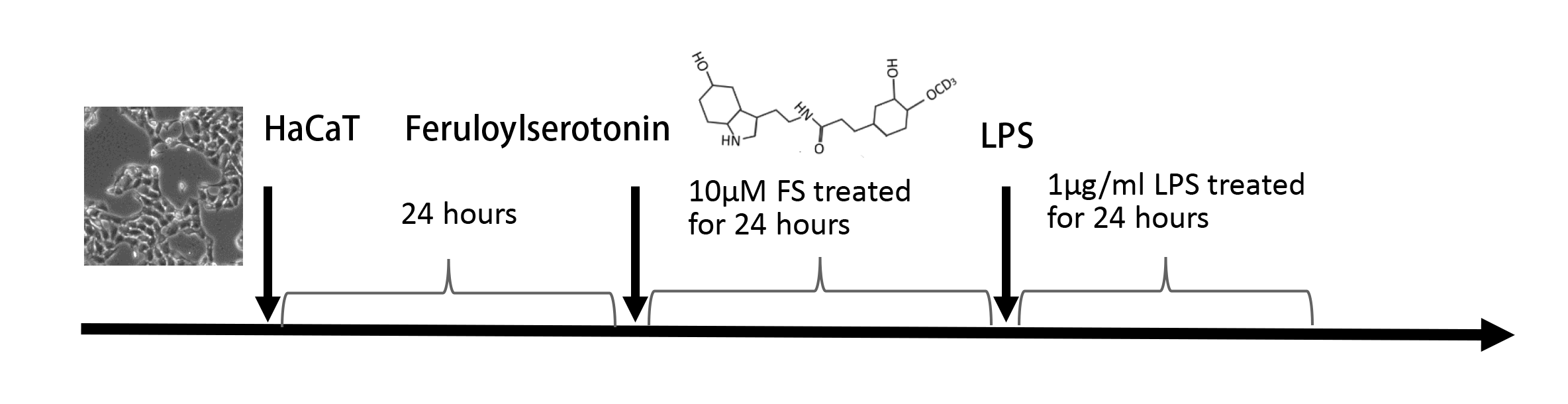

Supplement: Supplementary file 1 [file molecules-24-03064-s001.zip › Supplementary File/Figure S2.tif]

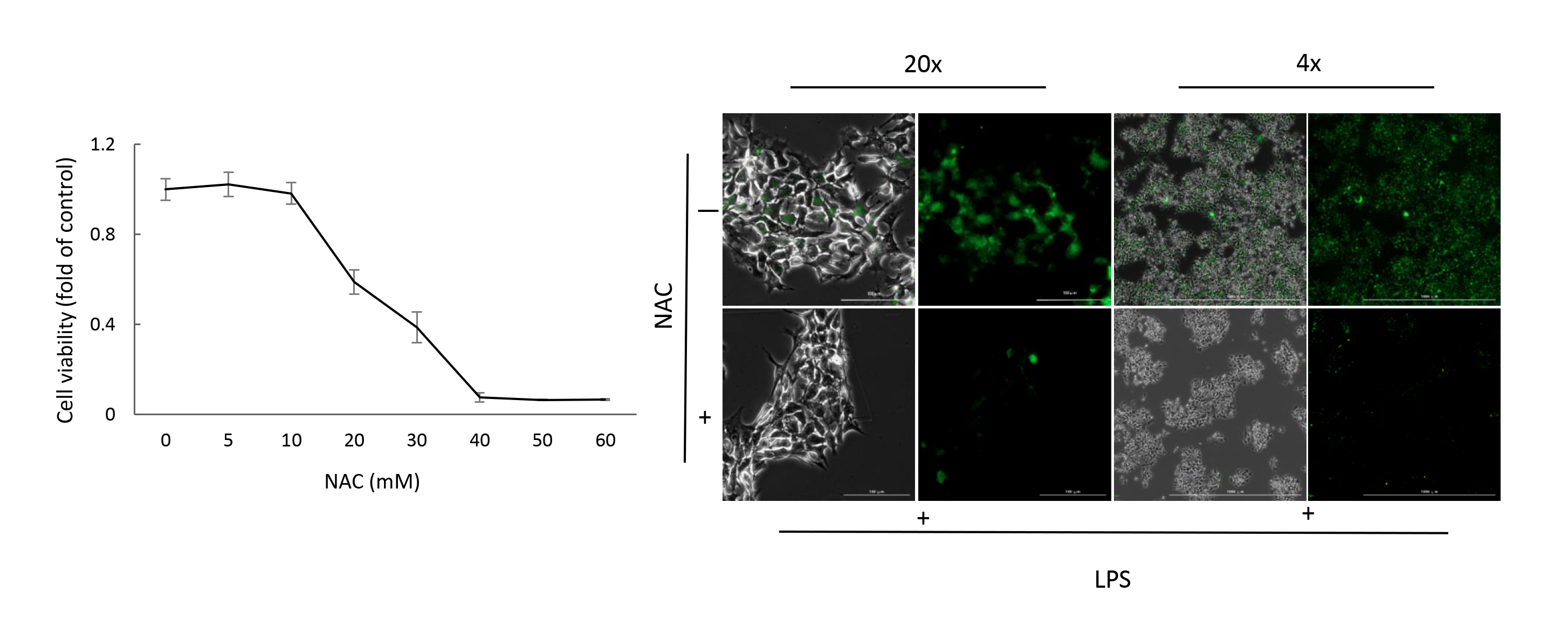

Supplement: Supplementary file 1 [file molecules-24-03064-s001.zip › Supplementary File/Figure S3.tif]

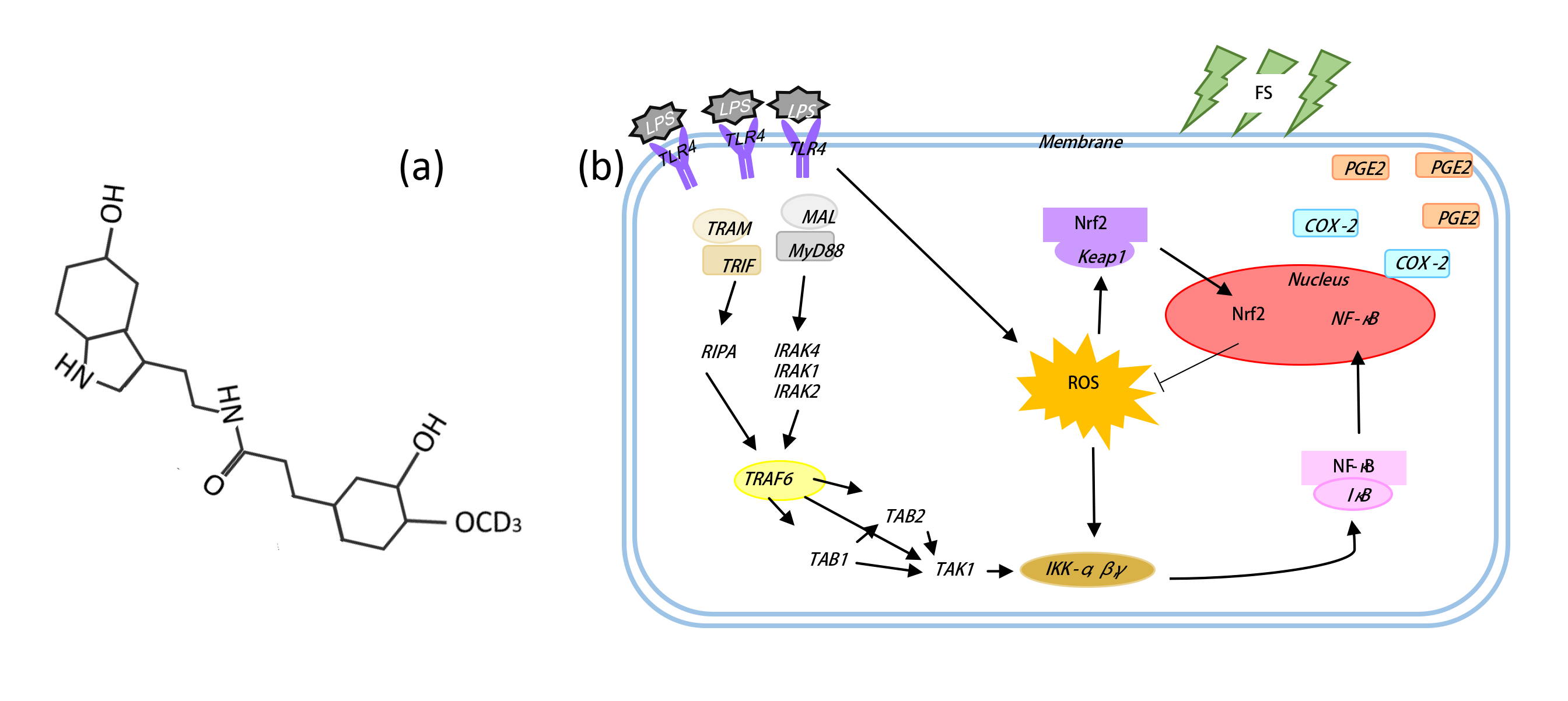

Supplement: Supplementary file 1 [file molecules-24-03064-s001.zip › Supplementary File/Figure S4.tif]
